# Evaluation of Gender Inequity in Thyroid Cancer Diagnosis: Differences by Sex in US Thyroid Cancer Incidence Compared With a Meta-analysis of Subclinical Thyroid Cancer Rates at Autopsy

**DOI:** 10.1001/jamainternmed.2021.4804

**Published:** 2021-08-30

**Authors:** Karissa LeClair, Katy J. L. Bell, Luis Furuya-Kanamori, Suhail A. Doi, David O. Francis, Louise Davies

**Affiliations:** 1 Geisel School of Medicine at Dartmouth, Hanover, New Hampshire; 2 Faculty of Medicine and Health, Sydney School of Public Health, The University of Sydney, New South Wales, Australia; 3 The University of Queensland Centre for Clinical Research, The University of Queensland, Herston, Australia; 4 Department of Population Medicine, College of Medicine, Qatar University Health, Qatar University, Doha, Qatar; 5 Wisconsin Surgical Outcomes Research and Division of Otolaryngology, Department of Surgery, University of Wisconsin, Madison; 6 The VA Outcomes Group, US Department of Veterans Affairs Medical Center, White River Junction, Vermont; 7 Section of Otolaryngology, Geisel School of Medicine at Dartmouth, Hanover, New Hampshire; 8 The Dartmouth Institute for Health Policy & Clinical Practice, Lebanon, New Hampshire

## Abstract

**Importance:**

Thyroid cancer is more common in women than in men, but the associated causes of these differences are not fully understood.

**Objective:**

To compare sex-specific thyroid cancer rates in the US to the prevalence of subclinical thyroid cancer at autopsy.

**Data Sources:**

Data on thyroid cancer incidence and mortality by sex among US adults (≥18 years) were extracted from the National Cancer Institute’s Surveillance, Epidemiology, and End Results Program (SEER) data for 1975 to 2017. Embase, PubMed, and Web of Science databases were searched for studies on the prevalence of subclinical thyroid cancer at autopsy of men and women, from inception to May 31, 2021.

**Study Selection:**

The Preferred Reporting Items for Systematic Reviews and Meta-analyses (PRISMA) reporting guideline was used to perform a systematic search for articles reporting the prevalence of subclinical thyroid cancer in autopsy results of both women and men. Of 101 studies identified, 8 studies containing 12 data sets met inclusion criteria; ie, they examined the whole thyroid gland, stated the number of thyroids examined, and reported results by sex. Excluded studies reported thyroid cancer in Japan after the atomic bombs or Chernobyl after the nuclear disaster; did not examine the whole thyroid gland or had incomplete information on thyroid examination methods; or did not report rates by sex.

**Data Extraction and Synthesis:**

Thyroid cancer incidence and mortality data by sex, histologic type, and tumor size were extracted from SEER. The inverse variance heterogeneity model was used to meta-analyze the prevalence and the odds ratio of subclinical thyroid cancer by sex from 8 studies (12 data sets) on thyroid cancer prevalence in autopsy results.

**Main Outcomes and Measures:**

Incidence and mortality of thyroid cancer, by histologic type and tumor size; prevalence of thyroid cancer in autopsy results.

**Results:**

In 2017, 90% of thyroid cancers diagnosed were papillary thyroid cancer (PTC) and in 2013 to 2017, the women to men incidence ratio for small (≤2 cm) PTC was 4.39:1. The incidence ratio approached 1:1 as cancer type lethality increased. The ratio of thyroid cancer mortality by gender was 1.02:1 and remained stable from 1992 to 2017. Results of the meta-analysis showed that the pooled autopsy prevalence of subclinical PTC was 14% in women (95% CI, 8%-20%) and 11% in men (95% CI, 5%-18%). The pooled odds ratio of subclinical PTC in women compared with men was 1.07 (95% CI, 0.80-1.42).

**Conclusions and Relevance:**

This cohort study and meta-analysis found that the belief that women get thyroid cancer more often than men is an oversimplification. The gender disparity is mostly confined to the detection of small subclinical PTCs, which are equally common in both sexes at autopsy but identified during life much more often in women than men. As the lethality of the cancer type increases, the ratio of detection by gender approaches 1:1. This phenomenon may be associated with gender differences in health care utilization and patterns of clinical thinking and can harm both women, who are subject to overdetection, and men, who may be at risk of underdetection.

## Key Points

**Question:**

How does thyroid cancer risk vary by sex?

**Findings:**

The cohort study in this comparison study found that the current ratio of small (≤2 cm) papillary thyroid cancer (PTC) incidence in women to men is 4.39:1, and the meta-analysis found that pooled odds ratio of subclinical PTC at autopsy in women compared with men is 1.07. The incidence rate ratio decreased as cancer lethality increased; and thyroid cancer mortality ratio by sex (women to men) was 1.02:1.

**Meaning:**

Gender differences in thyroid cancer are mostly confined to the detection of small PTCs; the incidence of more lethal types of thyroid cancer, thyroid cancer mortality, and subclinical cancer are similar by gender.

## Teaser

This cohort study and meta-analysis examines differences by sex in US thyroid cancer incidence ratios compared with subclinical thyroid cancer rates at autopsy to understand more about gender inequities in thyroid cancer diagnosis.

## Introduction

In the late 1970s and early 1980s in the US, thyroid ultrasonography and needle biopsy were introduced and adopted for evaluation of thyroid nodules. Ultrasonography improved visualization and needle biopsy became especially valuable because through it physicians could better differentiate benign from malignant lesions—better than any radionuclide or other imaging technique then or since has been able to do.^1^ The goal of these technologies was to advance detection and diagnostic precision. However, beginning in the early 1990s in the US, new cases of thyroid cancer, which had been rare, more than tripled in incidence without showing any benefit in lower mortality.^2,3,4^ This phenomenon has been mirrored in many other countries around the world.^2,3,5,6^ In the US, thyroid cancer is now the fifth most commonly diagnosed cancer in women and the ninth most common in men, and is projected to be the most common cancer diagnosed in people 15 to 29 years old in 2021.^7^

Papillary thyroid cancers (PTCs) often exist subclinically. Autopsy results indicate that approximately 11% of people have 1 or more PTC foci in the thyroid gland or in a nearby lymph node.^8,9,10^ These are individuals who died of other causes, not knowing that they had thyroid cancer. The rapidly rising incidence rates of thyroid cancer have been attributed to detection of this subclinical disease reservoir.^2^ Research has confirmed that the broad availability and use of ultrasonography to find nodules and guide needle biopsy procedures have correlated with rising detection rates.^2,11,12,13^ More detailed diagnostic practices in pathology departments have likely also contributed, along with new technology to aid surgeons in safely performing thyroid surgery.^14,15^ Safer surgery makes it easier to resolve questions about the level of threat that a thyroid nodule poses by surgically removing it when biopsy results are inconclusive.^14,15^ However, there are also concerns that there may be a concurrent true increase in the incidence of thyroid cancer, given that among patients presenting with advanced disease at diagnosis, there has been an increase in mortality.^4^ Detailed analyses of incidence and mortality patterns may help answer some of these questions, and an important place to begin is in examining differences by gender.

Thyroid cancer has always been more common in women than men, but the differences have become even more pronounced in recent years as incidence has increased in the US and many other countries. To date, investigations of biological causes of gender-specific differences in thyroid cancer have been inconclusive.^3,16,17^ We analyzed US incidence and mortality data for women and men for the major thyroid cancer histologic types and tumor size categories and performed a meta-analysis by sex to investigate prevalence rates of subclinical thyroid cancer in the results of whole thyroid gland autopsy in individuals with no known thyroid disease.

## Methods

### Cohort Study of Thyroid Cancer Incidence and Mortality

#### Data Sources

Thyroid cancer incidence data for 1975 to 2017 were extracted from the National Cancer Institute’s Surveillance, Epidemiology, and End Results Program–9 Registries (SEER; https://seer.cancer.gov/), which collects and provides population-based data on cancer incidence in the US from 1975 to the present, including histologic type and initial treatment. For analyses of tumor size, we used January 1, 1983, as the start date because that was when SEER started collecting these data.

Data on annual thyroid cancer mortality (all histologic types combined) for 1975 to 2017 were extracted from the Center for Disease Control and Prevention’s National Vital Statistics System (NVSS), which collects data on the underlying cause of death reported on death certificates filed by each state. Estimates of thyroid cancer mortality by histologic type from 1992 to 2017 were obtained from SEER using the incidence-based mortality data. Patients whose incident cancers are captured in SEER are followed-up until death, regardless of their place of residence at the time of death.

#### Thyroid Cancer Definitions

All thyroid cancer incidence and mortality rates were reported per 100 000 individuals and were age-adjusted to the year 2000 US standard population. Included cancer cases were defined as those with “thyroid” as the site of origin (*International Classification of Diseases for Oncology, 3rd Revision,* code 73.9). By convention, the SEER system includes in the cohort only the *first matching record*; therefore, if a person has had more than 1 cancer, their case is only included if the cancer of interest was the first cancer (ie, a patient diagnosed with a thyroid cancer after previously having had a lung cancer would not be included). Detailed definitions of the groupings for histologic type, cancer stage, and tumor size are described in eTable 1 in the Supplement. Cancers with missing stage data were excluded from both the numerator and the denominator. Cancer tumor size was captured using 3 different variables during the study period as described in eTable 1 in the Supplement. Despite changing variables over time, all coding rules for tumor size measurement have remained the same, so comparisons across variables were expected to be valid. Cases were excluded if tumor size data were missing or if they were listed as “no mass; no tumor found.”

#### Statistical Analysis

Thyroid cancer incidence and mortality rates and 95% CIs were calculated using SEER*Stat, version 8.3.9 (Surveillance Research Program, National Cancer Institute). Incidence trends in women and men were examined over time, including subanalyses by histologic type, tumor stage, and tumor size. To minimize variations owing to small case numbers, incidence rates by histologic type were averaged across 5-year intervals. To calculate incidence ratios of women to men (W:M) and 95% CIs, the average rate in women was divided by the average rate in men for a given time period.

Overall annual thyroid cancer mortality rates in 1975 to 2017 were calculated using data from the NVSS. Histologic-type–specific thyroid cancer mortality was calculated in SEER using incidence-based mortality data from 1992 to 2017. The choice to use 1992 to begin the incidence-based mortality estimates was based on the analysis that by 1992, the incidence-based mortality rate in SEER approximated that of the NVSS, indicating the SEER data source had matured—it approximated US thyroid cancer mortality. To calculate W:M mortality ratios, the average mortality rate in women was divided by the average mortality rate in men for a given time period.

### Meta-analysis of Subclinical Thyroid Cancer Prevalence at Autopsy

A systematic search following the Preferred Reporting Items for Systematic Reviews and Meta-analyses (PRISMA) reporting guideline^18^ was performed to identify articles reporting the prevalence of subclinical thyroid cancer in autopsy results of both women and men. We reproduced the search strategy described in the 2016 meta-analysis by Furuya-Kanamori and colleagues^8^ and updated it through May 31, 2021. Methods are described fully in the original article and in the eMethods in the Supplement.

#### Data Sources

Autopsy prevalence data for subclinical thyroid cancer were derived from a systematic search of Embase, PubMed, and Web of Science databases from inception to May 31, 2021. A manual search was also performed of articles cited within each included text, articles that cited included studies, and those identified by the “related articles” function in Google Scholar. Details of the search strategy and results from the updated search are available in the eMethods of the Supplement.

#### Study Inclusion Criteria

To meet the inclusion criteria, a study had to have examined the whole thyroid gland, as well as have reported the number of thyroid glands examined and the subclinical thyroid cancer autopsy prevalence for both women and men. A study was excluded if it did not report rates by sex; if its information on the methods used to examine the thyroid gland were incomplete; if the participants had a known history of thyroid pathology at the time of death; or if its findings were related to the atomic bombing of Japan or the Chernobyl nuclear disaster.

#### Study Quality Assessment

The studies were assessed using the Hoy and colleagues^19^ quality assessment checklist for prevalence studies,^20^ which consists of 9 safeguards, 4 of which are for external validity and 5 for internal validity. While external validity safeguards are not usually considered to safeguard against bias, an exception is made for prevalence and incidence studies because external validity safeguards do not affect the bias of the prevalence results.^19^

#### Statistical Analysis of Included Studies

Studies were independently reviewed for eligibility by 2 reviewers (L.D. and K.L.) using the same inclusion and exclusion criteria as the 2016 systematic review^8^ (eFigure 1 in the Supplement). Data were extracted from the articles meeting inclusion criteria for this analysis from May 1, 2019, to May 31, 2021. Statistical analyses were performed using Rayyan QCRI, version 0.1.0 (Qatar Computing Research Institute); *P* values were 2-tailed and statistical significance was defined as *P* < .05.

The pooled prevalence of subclinical thyroid cancer was estimated by sex. The double arcsine square root transformation was used to stabilize the variance of the prevalence.^21^ For ease of interpretation, the results were reported after back-transformation. Given that differences in population characteristics (eg, age, proportion of women, comorbidities) were expected across studies, the pooled prevalence odds ratio was estimated to identify differences in subclinical thyroid cancer prevalence by sex within studies.^22^ Both analyses were conducted using the inverse variance heterogeneity model of meta-analysis.^23^ For the prevalence odds ratio, the Doi plot and LFK index were used to examine the association between estimated effects and a measure of study size. Symmetry (no association) usually implies a lower likelihood of reporting biases (eg, publication bias).^24^

### Ethics Approval

The US Department of Veterans Affairs Institutional Review Board for Northern New England has deemed studies using deidentified, publicly available data (eg, published autopsy studies and SEER data) to be exempt from review and to not require informed consent in accordance with the Common Rule. Permission to access and use the SEER data was obtained through a data use agreement with the SEER program.

## Results

### Thyroid Cancer Incidence and Mortality

**Figure 1.**
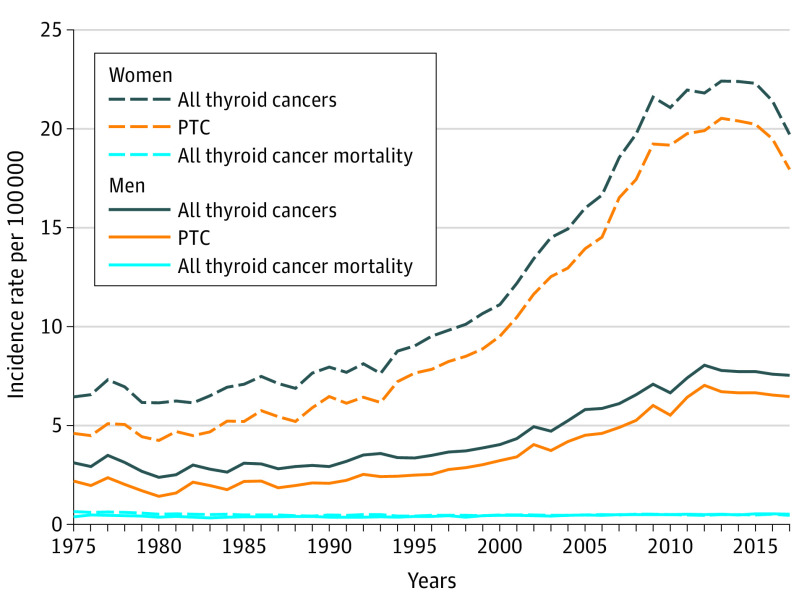
Thyroid Cancer Incidence and Mortality Trends by Sex, 1975 to 2017 PTC denotes papillary thyroid cancer.

Thyroid cancer diagnoses began to rise rapidly in the 1990s, peaking at 22.4 cases per 100 000 women and 7.8 per 100 000 men in 2013 (Figure 1), an increase driven by PTC diagnoses. During 1975 to 1989, papillary was the histologic type of 75% to 80% of thyroid cancers diagnosed, and by the period from 2010 to 2017, it was 90%. Women were affected more than men by this trend: between 1975 and 2017, PTC incidence grew by 13.3 cases per 100 000 women (from 4.6 to 17.9 cases or 389%) compared with 4.3 cases per 100 000 men (from 2.2 to 6.5 cases or 295%).

Table 1 illustrates the disparity in diagnosis rates by gender; the greatest disparity was for small PTCs. Between 1983 and 2017, women were diagnosed with small localized PTCs at a ratio of 4.28:1 compared with men. The ratio decreased when larger and higher stage PTCs were included (2.41:1). Underlying rates used to calculate the ratios are shown in eTables 2A and 2B in the Supplement.

**Table 1.** Women to Men Ratios of Thyroid Cancer Incidence, Mortality, and Prevalence

Measure and years	Women to men ratio (95%CI)
Incidence ratio, 1983-2017	
All thyroid cancers	2.75:1 (2.73-2.76)
PTC	2.97:1 (2.95-2.99)
Small (≤2 cm), localized, papillary	4.28:1 (3.96-4.65)
All other size and stages, papillary	2.41:1 (2.30-2.52)
Mortality ratio, 1992-2017	
All thyroid cancers	1.02:1 (1.015-1.020)
PTC	0.96:1 (0.95-0.97)
Prevalence (autopsy) odds ratio, 1975-2017	
Subclinical PTC	1.07:1 (0.80-1.42)

Abbreviation: PTC, papillary thyroid cancer.

Source: Surveillance, Epidemiology, and End Results Program (https://seer.cancer.gov/), SEER*Stat Database, 9 Registries, Surveillance Research Program, National Cancer Institute, released April 2021 based on the November 2020 submission.

Overall, annual thyroid cancer mortality data from the NVSS for 1975 to 2017 ranged from 0.4 to 0.6 per 100 000 women and 0.3 to 0.6 per 100 000 men. From 1992 to 2017, the average annual W:M mortality ratio was 1.02:1 (Table 1). We used the SEER incidence-based mortality data for 1992 to 2017 to obtain more detailed estimates of thyroid cancer mortality by histologic type. Mortality for thyroid cancers categorized as papillary ranged from 0.11 to 0.29 per 100 000 for women and 0.12 to 0.28 per 100 000 for men, with a W:M ratio during that time period of 0.96:1. Evaluating cancers for the years when data on tumor size and stage became available and consistent (SEER staging methodology changed in 2016, and estimates for 2016-2017 are too unstable/small to report), the incidence-based mortality rate for small (≤2 cm) localized PTCs was 0.003 per 100 000 between 1992 and 2015. This compares to a mortality rate of 0.084 per 100 000 for all other PTCs, and 0.19 per 100 000 for all remaining thyroid cancer histologic types (medullary, anaplastic, and follicular) during the same time period.

**Figure 2.**
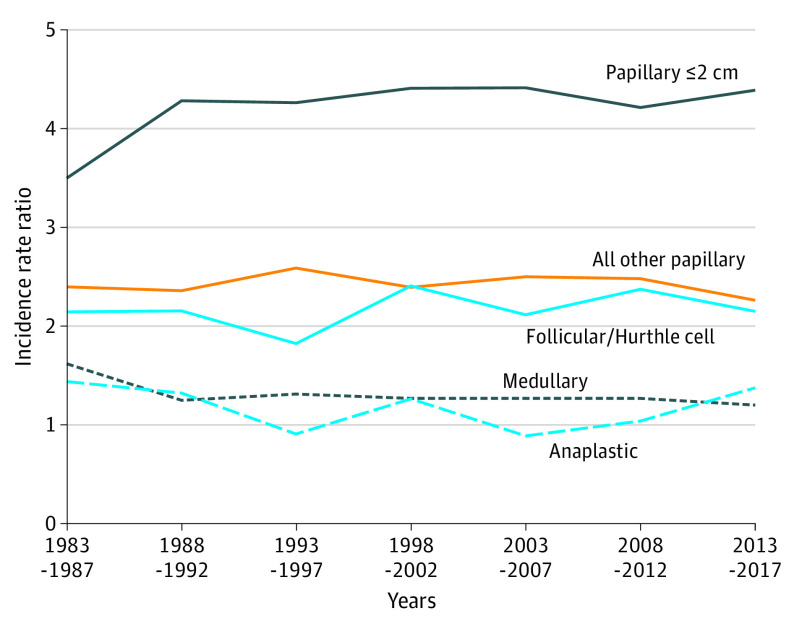
Women to Men Diagnosis Ratio of Thyroid Cancer by Type, 1983 to 2017

Figure 2 illustrates how detection ratios by sex compare when analyzed by thyroid cancer size and histologic type over time. Across 2 time periods, from 1983 to 1987 and 2013 to 2017, the W:M detection ratio for small localized PTCs started at 3.5:1 in 1983 and ended at 4.39:1 in 2017. The W:M ratios either remained stable or decreased over time for all other PTC sizes and stages, follicular and Hurthle cell, medullary, and anaplastic thyroid cancers. As the relative lethality of the cancer increased, the detection ratio moved closer to 1:1, with medullary and anaplastic cancers diagnosed more equally between sexes or slightly more in men during some time periods.

### Prevalence of Subclinical Thyroid Cancer

Table 2 summarizes the findings of the updated systematic review and meta-analysis for studies reporting the prevalence of PTC in autopsy results. Of the 35 original studies identified by the prior systematic review,^8^ 12 study populations in 8 studies^9,25,26,27,28,29,30,31^ were identified in which thyroid cancer prevalence was reported by sex and the whole thyroid gland was examined during the autopsy procedure. The updated search from November 1, 2015, to May 31, 2021, identified 3 articles for full-text review; however, 1 article did not report thyroid cancer cases by sex and the other 2 were conference abstracts whose authors did not respond to our requests for their data. No additional qualifying studies were identified by a manual search of references, citations, and related articles.

**Table 2.** Study Populations Included in a Meta-analysis of Thyroid Cancer Prevalence at Autopsy

Source	Country	Men		Women	
		Total cases	Subclinical papillary carcinoma, No. (%)	Total cases	Subclinical papillary carcinoma, No. (%)
Fukunaga and Yatani,^30^ 1975	Canada	62	3 (4.8)	38	3 (7.9)
Fukunaga and Yatani,^30^ 1975	Colombia	446	23 (5.2)	161	11 (6.8)
Fukunaga and Yatani,^30^ 1975	US	140	29 (20.7)	108	31 (28.7)
Fukunaga and Yatani,^30^ 1975	Japan	59	16 (27.1)	43	13 (30.2)
Fukunaga and Yatani,^30^ 1975	Poland	56	2 (3.6)	54	8 (14.8)
Seta and Takahashi,^31^ 1976	Japan	181	27 (14.9)	198	32 (16.2)
Harach et al,^29^ 1985	Finland	53	23 (43.4)	48	13 (27.1)
Komorowski and Hanson,^28^ 1988	US	86	3 (3.5)	52	2 (3.8)
Ottino et al,^9^ 1989	Argentina	59	8 (13.6)	41	3 (7.3)
Thorvaldsson et al,^27^ 1992	Iceland	160	12 (7.5)	39	2 (5.1)
Martinez-Tello et al,^26^ 1993	Spain	66	16 (24.2)	34	6 (17.6)
Neuhold et al,^25^ 2001	Austria	57	6 (10.5)	61	4 (6.6)

Of the 8 studies, 6 implemented all internal validity safeguards; however, all missed at least 1 external validity safeguard and 4 studies missed 2 or more. The most common missed safeguard was the autopsy service receiving individuals who closely represented the national population. A detailed assessment is reported in eTable 3 in the Supplement.

**Figure 3.**
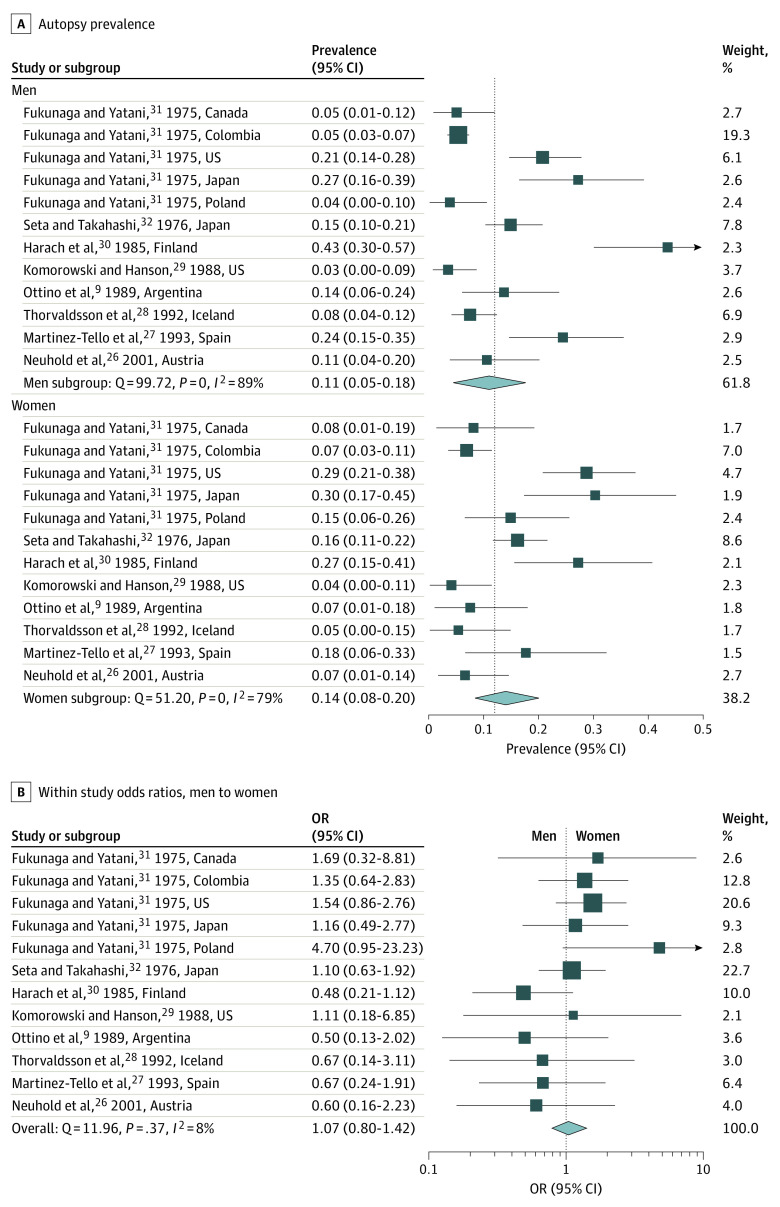
Meta-analysis for Autopsy Prevalence of Subclinical Papillary Thyroid Cancer by Sex The dotted line in part A depicts the overall meta-analysis estimate of prevalence, 0.12 (both sexes combined); in part B, it depicts an odds ratio of 1.0 (no difference between the sexes); and OR denotes odds ratio.

In the 12 pooled study populations (n = 2302; 877 women; 1425 men), the autopsy prevalence of subclinical PTC was 14.0% (95% CI, 8.4%-20.3%) in women and 10.8% (95% CI, 4.7%-17.9%) in men (Figure 3A). The test for interaction revealed that the difference in prevalence between women and men was not statistically significant (*P* = .49; Figure 3A), ie, there was no evidence of subgroup effect by sex.

Examining the distribution within the 8 individual studies, none had a difference in risk of subclinical PTC between sexes. The within-study prevalence odds ratio in women compared with men was 1.07 (95% CI, 0.80-1.42) indicating that the prevalence of subclinical PTC was similar between women and men (Figure 3B). The Doi plot was symmetrical (LFK index, −0.31) suggesting that the association between estimated effects and a measure of study size was similar to what might be expected to occur by chance (see eFigure 2 in the Supplement).

## Discussion

We are all taught in medical school that women get thyroid cancer more often than men. While technically true, we show in this study that this statement is an oversimplification. The gender disparity in thyroid cancer incidence in the US is mostly confined to the detection of small subclinical PTCs. As of 2017, women were diagnosed with small localized PTCs at a ratio of 4.39:1 compared with men. This meta-analysis of the prevalence of subclinical PTC at autopsy demonstrates no statistically detectable difference by sex: 14% in women (95% CI, 8%-20%) and 11% in men (95% CI, 5%-18%), with a prevalence odds ratio of 1.07 (95% CI, 0.80-1.42).

Among other thyroid cancer sizes and histologic types, as the lethality of the cancer type increased, the incidence rate ratio moved closer to 1:1. Medullary- and anaplastic-type thyroid cancers, which have 5-year survival rates of 89% and <10% respectively,^32,33^ are diagnosed only slightly more often in women than in men. The mortality ratio by sex is also near 1:1 (W:M ratio, 0.96:1 for PTC and 1.02:1 for all thyroid cancers).

These findings have important implications for all genders. Small PTCs, diagnosed over 4 times more frequently in women than men, are the type most likely to be differentially identified by diagnostic practices given the large reservoir of subclinical papillary cancers. Thyroid cancer incidence may be more common in women because of our patterns in clinical thinking—clinicians have been taught to look for it more often in this population. This self-perpetuating cycle of looking more often in women, and subsequently finding disease, has adverse effects for all genders. Women are more likely to have small subclinical cancers detected, for which the benefits of aggressive treatment are unclear and potential harms and adverse effects are substantial, given what we know about the natural history and high likelihood of survival for early-stage PTC.^34^ Meanwhile, men are at risk of being diagnosed later than they might have otherwise been because thyroid cancer was placed lower in the differential diagnosis. The prior analysis of autopsy data^8^ has established that the underlying prevalence of subclinical thyroid cancer discovered at autopsy has not changed over time.

It is not just that we look for thyroid cancer more often in women than men, however. There are also differences in health care utilization patterns. Research shows that women seek and utilize health care more often than men do, even when controlling for reproductive health visits.^35,36^ This generates more opportunities to find thyroid nodules and diagnose thyroid cancer. Among the Medicare population, the use of thyroid ultrasonography as a first imaging study increased from 2002 to 2013, with its use associated with patients who were women.^11^ Women are also more likely than men to be referred for thyroid ultrasonography by general practitioners for poorly-defined concerns (eg, fatigue, menstrual disturbance).^37^ The use of thyroid ultrasonography as a tool to evaluate ill-defined symptoms can lead to the detection of small thyroid cancers that do not explain the patient’s presenting symptom—a pathway to detection that has been termed the diagnostic cascade.^38^

Some experts have proposed that thyroid cancer is more common in women than men because of reproductive and hormonal factors. Weak results and discrepant data have characterized numerous in vitro and animal studies probing the link between estrogen and thyroid cancer, with reviews concluding that a link has not been established, although some observations and mechanisms proposed show thyroid tissue sensitivity to estrogen and estrogen-like compounds.^17,39^ On a population level, some investigations have suggested a possible link between thyroid cancer risk and factors such as recent pregnancy, history of infertility, abnormal menstrual cycles, and history of breast cancer.^17,40,41,42,43^ However, a systematic review conducted in 2012 found that no reproductive or hormonal factors were consistently associated with thyroid cancer risk.^44^ Authors instead suggested that higher rates of thyroid cancer might be attributable to greater health care engagement and likelihood for nonspecific evaluations during care for other medical and reproductive conditions.^42,44^

### Study Strengths and Limitations

This present study has several strengths, including the use of SEER data which is the best source of high-quality, population-based cancer incidence and mortality data in the US. The meta-analysis techniques we used were rigorous; however, we do not know how or why the thyroid cancer cases captured in SEER were detected, which somewhat limits our ability to make inferences about subclinical detection vs symptomatic detection. Second, the autopsy studies were small and may not be representative of all populations. Meta-analysis and assessments of study quality, such as those we used, can overcome some of these limitations.

## Conclusions

Efforts to limit the detection of subclinical disease must be balanced with the need to optimize detection of thyroid cancers that are likely to be more aggressive. Recent refinements in treatment guidelines have been made to decrease overtreatment, but many of these small asymptomatic cancers are currently treated the same way as those that come to attention because of their larger size and/or more rapid growth. As of 2017, 88% of patients with thyroid cancer were still undergoing total thyroidectomy for small cancers, although the guidelines suggested that hemithyroidectomy was acceptable.^45,46,47,48,49^

The findings of the present study suggest that women may benefit from guidelines aimed at promoting more focused use of thyroid imaging. While men are also at risk of having subclinical disease detected, they may be diagnosed with clinical disease at later stages because they present for health care less often and may have thyroid cancer placed lower in the differential than women with similar symptoms. The gender-specific knowledge gained by this study should inform careful selection of patients for thyroid evaluation and examination to ensure that men and women are both receiving appropriate care.

These study findings demonstrate that the statement that thyroid cancer is more common in women than in men has been oversimplified. Small PTCs are detected much more often in women than in men, but the prevalence of these subclinical cancers at autopsy is not different. As the lethality of the cancer type increases, the women-to-men incidence ratio approaches 1:1. Mortality rates are also similar between women and men. Systemic efforts should be made to limit the harms that can come from the detection and aggressive treatment of small low-risk PTCs, particularly in women. At the same time, we should be aware of and attend to the more aggressive thyroid cancers that are nearly equally as likely to present in both men and women.

## Notes

Supplement. **eTable 1.** Case Definitions and Data Sources, Thyroid Cancer (ICDO-03, code 73.9) Incidence and Mortality Data **eFigure 1.** PRISMA Flow Diagram of Study Selection for Quantitative Synthesis **eMethods.** Autopsy Meta-analysis Search Strategy and Results **eTable 2a.** Incidence per 100 000 People for Thyroid Cancer During 1975–2017, SEER 9 Data **eTable 2b.** Mortality per 100 000 People for Thyroid Cancer During 1975–2017, Using National Center for Vital Statistics data (1975-2017, all thyroid cancer) and SEER Incidence-Based Mortality Data (1992-2017, papillary thyroid cancer) **eTable 3.** Quality Assessment of the Included Studies in the Meta-analysis **eFigure 2.** Doi Plot and LFK Index for Assessment of Publication Bias

## References

[1] Rojeski MT, Gharib H. Nodular thyroid disease: evaluation and management. N Engl J Med. 1985;313(7):428-436. doi:10.1056/NEJM198508153130707 3894966

[2] Davies L, Welch HG. Increasing incidence of thyroid cancer in the United States, 1973-2002. JAMA. 2006;295(18):2164-2167. doi:10.1001/jama.295.18.2164 16684987

[3] Davies L, Welch HG. Current thyroid cancer trends in the United States. JAMA Otolaryngol Head Neck Surg. 2014;140(4):317-322. doi:10.1001/jamaoto.2014.1 24557566

[4] Lim H, Devesa SS, Sosa JA, Check D, Kitahara CM. Trends in thyroid cancer incidence and mortality in the United States, 1974-2013. JAMA. 2017;317(13):1338-1348. doi:10.1001/jama.2017.2719 28362912 PMC8216772

[5] Li M, Dal Maso L, Vaccarella S. Global trends in thyroid cancer incidence and the impact of overdiagnosis. Lancet Diabetes Endocrinol. 2020;8(6):468-470. doi:10.1016/S2213-8587(20)30115-7 32445733

[6] Vaccarella S, Dal Maso L, Laversanne M, Bray F, Plummer M, Franceschi S. The impact of diagnostic changes on the rise in thyroid cancer incidence: a population-based study in selected high-resource countries. Thyroid. 2015;25(10):1127-1136. doi:10.1089/thy.2015.0116 26133012

[7] National Cancer Institute. Surveillance, Epidemiology and End Results Program. Cancer stat facts: thyroid cancer; 2020. Accessed December 6, 2020. https://seer.cancer.gov/statfacts/html/thyro.html

[8] Furuya-Kanamori L, Bell KJL, Clark J, Glasziou P, Doi SAR. Prevalence of differentiated thyroid cancer in autopsy studies over 6 decades: a meta-analysis. J Clin Oncol. 2016;34(30):3672-3679. doi:10.1200/JCO.2016.67.7419 27601555

[9] Ottino A, Pianzola HM, Castelletto RH. Occult papillary thyroid carcinoma at autopsy in La Plata, Argentina. Cancer. 1989;64(2):547-551. doi:10.1002/1097-0142(19890715)64:2<547::AID-CNCR2820640232>3.0.CO;2-N 2736500

[10] Sampson RJ, Oka H, Key CR, Buncher CR, Iijima S. Metastases from occult thyroid carcinoma: an autopsy study from Hiroshima and Nagasaki, Japan. Cancer. 1970;25(4):803-811. doi:10.1002/1097-0142(197004)25:4<803::AID-CNCR2820250409>3.0.CO;2-P 5443104

[11] Haymart MR, Banerjee M, Reyes-Gastelum D, Caoili E, Norton EC. Thyroid ultrasonography and the increase in diagnosis of low-risk thyroid cancer. J Clin Endocrinol Metab. 2019;104(3):785-792. doi:10.1210/jc.2018-01933 30329071 PMC6456891

[12] Udelsman R, Zhang Y. The epidemic of thyroid cancer in the United States: the role of endocrinologists and ultrasounds. Thyroid. 2014;24(3):472-479. doi:10.1089/thy.2013.0257 23937391 PMC3949447

[13] Cronan JJ. Thyroid nodules: is it time to turn off the US machines? Radiology. 2008;247(3):602-604. doi:10.1148/radiol.2473072233 18487528

[14] Grodski S, Brown T, Sidhu S, . Increasing incidence of thyroid cancer is due to increased pathologic detection. Surgery. 2008;144(6):1038-1043. doi:10.1016/j.surg.2008.08.023 19041015

[15] Higgins TS, Gupta R, Ketcham AS, Sataloff RT, Wadsworth JT, Sinacori JT. Recurrent laryngeal nerve monitoring versus identification alone on post-thyroidectomy true vocal fold palsy: a meta-analysis. Laryngoscope. 2011;121(5):1009-1017. doi:10.1002/lary.21578 21520117

[16] Filetti S, Tuttle M, Leboulleux S, Alexander EK. Nontoxic diffuse goiter, nodular thyroid disorders, and thyroid malignancies. In: Melmed S, Auchus RJ, Goldfine AB, Koenig RJ, Rosen CJ, eds. Williams Textbook of Endocrinology. 14th ed. Elsevier; 2020.

[17] Moleti M, Sturniolo G, Di Mauro M, Russo M, Vermiglio F. Female reproductive factors and differentiated thyroid cancer. Front Endocrinol (Lausanne). 2017;8:111. doi:10.3389/fendo.2017.00111 28588554 PMC5440523

[18] Moher D, Liberati A, Tetzlaff J, Altman DG; PRISMA Group. Preferred reporting items for systematic reviews and meta-analyses: the PRISMA statement. BMJ. 2009;339:b2535. doi:10.1136/bmj.b2535 19622551 PMC2714657

[19] Hoy D, Brooks P, Woolf A, . Assessing risk of bias in prevalence studies: modification of an existing tool and evidence of interrater agreement. J Clin Epidemiol. 2012;65(9):934-939. doi:10.1016/j.jclinepi.2011.11.014 22742910

[20] Furuya-Kanamori L, Xu C, Hasan SS, Doi SA. Quality versus risk-of-bias assessment in clinical research. J Clin Epidemiol. 2021;129:172-175. doi:10.1016/j.jclinepi.2020.09.044 33422267

[21] Barendregt JJ, Doi SA, Lee YY, Norman RE, Vos T. Meta-analysis of prevalence. J Epidemiol Community Health. 2013;67(11):974-978. doi:10.1136/jech-2013-203104 23963506

[22] Doi SA, Furuya-Kanamori L, Xu C, Lin L, Chivese T, Thalib L. Questionable utility of the relative risk in clinical research: a call for change to practice. J Clin Epidemiol. 2020;S0895-4356(20)31171-9. 33171273 10.1016/j.jclinepi.2020.08.019

[23] Doi SA, Barendregt JJ, Khan S, Thalib L, Williams GM. Advances in the meta-analysis of heterogeneous clinical trials I: The inverse variance heterogeneity model. Contemp Clin Trials. 2015;45(Pt A):130-138. doi:10.1016/j.cct.2015.05.009 26003435

[24] Furuya-Kanamori L, Barendregt JJ, Doi SAR. A new improved graphical and quantitative method for detecting bias in meta-analysis. Int J Evid Based Health. 2018;16(4):195-203. doi:10.1097/XEB.0000000000000141 29621038

[25] Neuhold N, Kaiser H, Kaserer K. Latent carcinoma of the thyroid in Austria: a systematic autopsy study. Endocr Pathol. 2001;12(1):23-31. doi:10.1385/EP:12:1:23 11478265

[26] Martinez-Tello FJ, Martinez-Cabruja R, Fernandez-Martin J, Lasso-Oria C, Ballestin-Carcavilla C. Occult carcinoma of the thyroid: a systematic autopsy study from Spain of 2 series performed with 2 different methods. Cancer. 1993;71(12):4022-4029. doi:10.1002/1097-0142(19930615)71:12<4022::AID-CNCR2820711236>3.0.CO;2-O 8508367

[27] Thorvaldsson SE, Tulinius H, Björnsson J, Bjarnason O. Latent thyroid carcinoma in Iceland at autopsy. Pathol Res Pract. 1992;188(6):747-750. doi:10.1016/S0344-0338(11)80172-X 1437838

[28] Komorowski RA, Hanson GA. Occult thyroid pathology in the young adult: an autopsy study of 138 patients without clinical thyroid disease. Hum Pathol. 1988;19(6):689-696. doi:10.1016/S0046-8177(88)80175-8 3378788

[29] Harach HR, Franssila KO, Wasenius VM. Occult papillary carcinoma of the thyroid–a “normal” finding in Finland: a systematic autopsy study. Cancer. 1985;56(3):531-538. doi:10.1002/1097-0142(19850801)56:3<531::AID-CNCR2820560321>3.0.CO;2-3 2408737

[30] Fukunaga FH, Yatani R. Geographic pathology of occult thyroid carcinomas. Cancer. 1975;36(3):1095-1099. doi:10.1002/1097-0142(197509)36:3<1095::AID-CNCR2820360338>3.0.CO;2-9 1182663

[31] Seta K, Takahashi S. Thyroid carcinoma. Int Surg. 1976;61(20):541-544. 977240

[32] Randle RW, Balentine CJ, Leverson GE, . Trends in the presentation, treatment, and survival of patients with medullary thyroid cancer over the past 30 years. Surgery. 2017;161(1):137-146. doi:10.1016/j.surg.2016.04.053 27842913 PMC5164945

[33] Lin B, Ma H, Ma M, . The incidence and survival analysis for anaplastic thyroid cancer: a SEER database analysis. Am J Transl Res. 2019;11(9):5888-5896. 31632557 PMC6789224

[34] Davies L, Welch HG. Thyroid cancer survival in the United States: observational data from 1973 to 2005. Arch Otolaryngol Head Neck Surg. 2010;136(5):440-444. doi:10.1001/archoto.2010.55 20479371

[35] Bertakis KD, Azari R. Patient gender differences in the prediction of medical expenditures. J Womens Health (Larchmt). 2010;19(10):1925-1932. doi:10.1089/jwh.2009.1448 20831429

[36] Wang Y, Hunt K, Nazareth I, Freemantle N, Petersen I. Do men consult less than women? an analysis of routinely collected UK general practice data. BMJ Open. 2013;3(8):e003320. doi:10.1136/bmjopen-2013-003320 23959757 PMC3753483

[37] Germano A, Schmitt W, Almeida P, Mateus-Marques R, Leite V. Ultrasound requested by general practitioners or for symptoms unrelated to the thyroid gland may explain higher prevalence of thyroid nodules in females. Clin Imaging. 2018;50:289-293. doi:10.1016/j.clinimag.2018.05.003 29738997

[38] Davies L, Ouellette M, Hunter M, Welch HG. The increasing incidence of small thyroid cancers: where are the cases coming from? Laryngoscope. 2010;120(12):2446-2451. doi:10.1002/lary.21076 21108428

[39] Rahbari R, Zhang L, Kebebew E. Thyroid cancer gender disparity. Future Oncol. 2010;6(11):1771-1779. doi:10.2217/fon.10.127 21142662 PMC3077966

[40] Horn-Ross PL, Canchola AJ, Ma H, Reynolds P, Bernstein L. Hormonal factors and the risk of papillary thyroid cancer in the California Teachers Study cohort. Cancer Epidemiol Biomarkers Prev. 2011;20(8):1751-1759. doi:10.1158/1055-9965.EPI-11-0381 21791618 PMC3288117

[41] Cao Y, Wang Z, Gu J, . Reproductive factors but not hormonal factors associated with thyroid cancer risk: a systematic review and meta-analysis. Biomed Res Int. 2015;2015:103515. doi:10.1155/2015/103515 26339585 PMC4538312

[42] Zamora-Ros R, Rinaldi S, Biessy C, . Reproductive and menstrual factors and risk of differentiated thyroid carcinoma: the EPIC study. Int J Cancer. 2015;136(5):1218-1227. doi:10.1002/ijc.29067 25041790

[43] Nielsen SM, White MG, Hong S, . The breast-thyroid cancer link: a systematic review and meta-analysis. Cancer Epidemiol Biomarkers Prev. 2016;25(2):231-238. doi:10.1158/1055-9965.EPI-15-0833 26908594 PMC4770576

[44] Peterson E, De P, Nuttall R. BMI, diet and female reproductive factors as risks for thyroid cancer: a systematic review. PLoS One. 2012;7(1):e29177. doi:10.1371/journal.pone.0029177 22276106 PMC3261873

[45] James BC, Timsina L, Graham R, Angelos P, Haggstrom DA. Changes in total thyroidectomy versus thyroid lobectomy for papillary thyroid cancer during the past 15 years. Surgery. 2019;166(1):41-47. doi:10.1016/j.surg.2019.01.007 30904172

[46] Ullmann TM, Gray KD, Stefanova D, . The 2015 American Thyroid Association guidelines are associated with an increasing rate of hemithyroidectomy for thyroid cancer. Surgery. 2019;166(3):349-355. doi:10.1016/j.surg.2019.03.002 31056200

[47] Grant EG, Tessler FN, Hoang JK, . Thyroid ultrasonography reporting lexicon: white paper of the ACR Thyroid Imaging, Reporting and Data System (TIRADS) Committee. J Am Coll Radiol. 2015;12(12 Pt A):1272-1279. doi:10.1016/j.jacr.2015.07.011 26419308

[48] Hoang JK, Langer JE, Middleton WD, . Managing incidental thyroid nodules detected on imaging: white paper of the ACR Incidental Thyroid Findings Committee. J Am Coll Radiol. 2015;12(2):143-150. doi:10.1016/j.jacr.2014.09.038 25456025

[49] Haugen BR, Alexander EK, Bible KC, . 2015 American Thyroid Association management guidelines for adult patients with thyroid nodules and differentiated thyroid cancer: the American Thyroid Association Guidelines Task Force on Thyroid Nodules and Differentiated Thyroid Cancer. Thyroid. 2016;26(1):1-133. doi:10.1089/thy.2015.0020 26462967 PMC4739132

