# Supplementary material for: Evaluation of Gender Inequity in Thyroid Cancer Diagnosis: Differences by Sex in US Thyroid Cancer Incidence Compared With a Meta-analysis of Subclinical Thyroid Cancer Rates at Autopsy
Source: JAMA Intern Med. 2021 Aug 30;181(10):1–8. doi: 10.1001/jamainternmed.2021.4804 (PMC8406211; doi:10.1001/jamainternmed.2021.4804)
Supplement: Supplement. — eTable 1. Case Definitions and Data Sources, Thyroid Cancer (ICDO-03, code 73.9) Incidence and Mortality Data eFigure 1. PRISMA Flow Diagram of Study Selection for Quantitative Synthesis eMethods. Autopsy Meta-analysis Search Strategy and Results eTable 2a. Incidence per 100 000 People for Thyroid Cancer During 1975–2017, SEER 9 Data eTable 2b. Mortality per 100 000 People for Thyroid Cancer During 1975–2017, Using National Center for Vital Statistics data (1975-2017, all thyroid cancer) and SEER Incidence-Based Mortality Data (1992-2017, papillary thyroid cancer) eTable 3. Quality Assessment of the Included Studies in the Meta-analysis eFigure 2. Doi Plot and LFK Index for Assessment of Publication Bias [file jamainternmed-e214804-s001.pdf]

Supplemental Online Content

LeClair K, Bell KJL, Furuya-Kanamori L, Doi SA, Francis DO, Davies L. Evaluation of gender inequity in thyroid cancer diagnosis: differences by sex in US thyroid cancer incidence compared with a meta-analysis of subclinical thyroid cancer rates at autopsy. Published online August 30, 2021. *JAMA Intern Med*. doi:10.1001/jamainternmed.2021.4804

eTable 1. Case Definitions and Data Sources, Thyroid Cancer (ICDO-03, code 73.9) Incidence and Mortality Data

eFigure 1. PRISMA Flow Diagram of Study Selection for Quantitative Synthesis

eMethods. Autopsy Meta-analysis Search Strategy and Results

eTable 2a. Incidence per 100 000 People for Thyroid Cancer During 1975–2017, SEER 9 Data

eTable 2b. Mortality per 100 000 People for Thyroid Cancer During 1975–2017, Using National Center for Vital Statistics data (1975-2017, all thyroid cancer) and SEER Incidence-Based Mortality Data (1992-2017, papillary thyroid cancer)

eTable 3. Quality Assessment of the Included Studies in the Meta-analysis

eFigure 2. Doi Plot and LFK Index for Assessment of Publication Bias

This supplemental material has been provided by the authors to give readers additional information about their work.

© 2021 LeClair et al. *JAMA Intern Med.*

eTable 1. **Case Definitions and Data Sources, Thyroid Cancer (ICD-03 73.9) Incidence and Mortality Data**	
**Histology Definitions – SeerStat Variable: 'ICD-03 Behavior Code'**	
Papillary Thyroid Cancer	ICD-03 8050, 8052, 8130, 8260, 8340-8344, 8450-8452
Follicular Thyroid Cancer	ICD-03 8330-8332, 8335
Hurthle Cell Thyroid Cancer (*combined for analysis with follicular type)*	ICD-03 8290
Medullary Thyroid Cancer	ICD-03 8345-8346, 8510
Anaplastic Thyroid Cancer	ICD-03 8021
**Stage Definitions – SeerStat Variable: 'SEER Historic Stage A' and 'SEER Combined Summary Stage (CSS) 2000**	
Localized	Confined to one lobe and/or isthmus; both lobes involved; thyroid gland capsule involved; multiple foci but confined to thyroid gland; through the capsule of gland but not beyond; 'localized, NOS'
Regional	Direct extension to pericapsular tissues, strap muscles, nerves (recurrent laryngeal, vagus), major blood vessels (carotid artery, thyroid artery or vein, jugular vein), soft tissues of neck, esophagus, larynx (including thyroid and cricoid cartilages), sternocleidomastoid muscle, or "fixed" to adjacent tissues. Lymph node involvement of anterior deep cervical (prelaryngeal, pretracheal, laterotracheal), internal jugular (subdigastric), retropharyngeal, or 'cervical NOS'
Distant	Direct extension to trachea*, mediastinal tissues, skeletal muscle (other than strap muscles or sternocleidomastoid), prevertebral fascia*(added in SEER CSS), bone, or other distant involvement. Lymph node involvement of submandibular (submaxillary), submental*, and other distant nodes
*Definition changes between SEER Historic Stage A and the SEER CSS 2000 (2004+)	• Direct extension to trachea was recategorized from distant to regional disease in SEER CSS<br>• Prevertebral fascia invasion was added into the definition of 'distant' in SEER CSS<br>• Lymph node involvement of submandibular (submaxillary), submental regions was recategorized from distant to regional in SEER CSS
**Size Definitions – SeerStat Variables:**<br>**'***Extent of Disease 4 – Size' for 1983-1987*<br>*'Extent of Disease 10 – Size' for 1988-2003*<br>*'Collaborative Stage Tumor Size' for 2004-2015*<br>*'Tumor Size Summary (2016+) for 2016-2017*	
1983-1987 less than 2 cm	001-020
1983-1987 more than 2 cm	021-097
1988-2003 less than 2 cm	001-020
1988-2003 more than 2 cm	021-990
2004-2015 less than 2 cm	001-020, 990-992
2004-2015 more than 2 cm	021-989, 993-995
2016-2017 less than 2 cm	001-020, 990
2016-2017 more than 2 cm	021- 989

© 2021 LeClair et al. *JAMA Intern Med.*

eMethods. Autopsy Meta-analysis Search Strategy and Results

The search strategy for the Furuya-Kanamori *et al* paper was built by a health librarian and included the following keywords and subject terms: “thyroid cancer,” “autopsy,” “prevalence,” and “incidental.” A second, different, title/subject term (Medical Subject Headings [MeSH] and Emtree) search was done in order to find studies performed before 1975. This was deemed necessary because of the lack of pre-1975 results found in the initial search, which we surmised was a result of the lack of abstracts in the early literature. To achieve a comprehensive evaluation of the published evidence, the systematic searches were combined with a forward and backward citation search, and the first 20 similar articles from PubMed for each of the articles included from the searches were retrieved. The search extended through December 31, 2015.

To update the search for this paper, the search was repeated to identify additional articles that might have come available after the prior paper was written. We limited the publication dates of the results in PubMed from November 2015 to May 31, 2021. We opted for a two-month overlap because the limit is for publication date not the date when the record was entered into the databases. This strategy would capture any articles that were published in late 2015 but were not in PubMed when at the time of the previous search. In Web of Science the results were limited to 2016 to May 31, 2021, because the search can only be refined by full year in this database.

The new search yielded 108 new articles, of which 300 were duplicates. After completing title and abstract review, three met the search criteria: one in Spanish and two conference abstracts. As one member of the team was a native Spanish speaker this was fully reviewed, but it did not report thyroid cancer cases by sex. Queries to authors of the conference abstracts went unanswered.

Data were Extracted and summarized in a spreadsheet, which included the following fields: Study Author(s), Publication Year, Sex Proportion, Country, Thyroid Examination Methodology, Number of Autopsies Performed, and Histologic Findings by Sex.

PubMed Search

"Thyroid Neoplasms"[MeSH] OR "Adenocarcinoma, Follicular"[MeSH] OR "Adenocarcinoma, Papillary"[MeSH] OR OPTC OR ((Thyroid[tiab] OR Follicular[tiab] OR Papillary[tiab] OR hurtle cell[tiab]) AND (cancer[tiab] OR cancers[tiab] OR carcinoma[tiab] OR carcinomas[tiab] OR Adenocarcinoma[tiab] OR Adenocarcinomas[tiab] neoplasm[tiab] OR neoplasms[tiab] OR nodule[tiab] OR nodules[tiab] OR tumor[tiab] OR tumour[tiab] OR Tumors[tiab] OR Tumours[tiab] OR cyst[tiab] OR cysts[tiab]))

AND

"Autopsy"[MeSH] OR "Autopsy"[tiab] OR "Autopsies"[tiab] OR "Postmortem"[tiab] OR Post-mortem[tiab] OR (Post[tiab] AND mortem[tiab])

AND

"Prevalence"[MeSH] OR "Epidemiology"[MeSH] OR "Prevalence"[tiab] OR "Prevalences"[tiab] OR Epidemiology[tiab] OR Epidemiological[tiab] OR Frequency[tiab]

AND

"Incidental Findings"[MeSH] OR Incidental[tiab] OR Unsuspected[tiab] OR Discovery[tiab] OR Discoveries[tiab] OR Findings[tiab] OR Finding[tiab] OR Occult[tiab] OR Hidden[tiab]

PubMed Title/MeSH Search (for Finding Article Before 1975)

"Thyroid Neoplasms"[MeSH] OR

(Thyroid[ti] AND (cancer[ti] OR cancers[ti] OR carcinoma[ti] OR carcinomas[ti] OR Tumor[ti] OR Tumors[ti] OR Tumor[ti] OR Tumours[ti] OR Neoplasm[ti] OR Neoplasms[ti]))

AND

("Autopsy"[MeSH] OR pathology[sh] OR Autopsy[ti] OR Autopsies[ti] OR Pathology[ti])

AND

(epidemiology[sh] OR Epidemiology[ti] OR Prevalence[ti] OR Patterns[ti] OR Cases[ti])

Embase Search
'thyroid cancer'/exp OR 'adenocarcinoma'/exp OR OPTC OR (Thyroid OR Follicular OR Papillary OR hurthle cell) AND (cancer OR cancers OR carcinoma OR carcinomas OR Adenocarcinoma OR Adenocarcinomas neoplasm OR neoplasms OR nodule OR nodules OR tumor OR tumour OR Tumors OR Tumours OR cyst OR cysts)

AND

'Autopsy'/exp OR Autopsy OR Autopsies OR Postmortem OR Post-mortem OR (Post AND mortem)

AND

‘prevalence'/exp OR 'epidemiology'/exp OR Prevalence OR Prevalences OR Epidemiology OR Epidemiological OR Frequency

AND

'incidental finding'/exp OR Incidental OR Unsuspected OR Discovery OR Discoveries OR Findings OR Finding OR Occult OR Hidden

Embase Title/Emtree Search (for Finding Articles Before 1975)
'thyroid cancer'/exp OR

((Thyroid:ti AND (cancer:ti OR cancers:ti OR carcinoma:ti OR carcinomas:ti OR Tumor:ti OR Tumors:ti OR Tumor:ti OR Tumours:ti OR Neoplasm:ti OR Neoplasms:ti)))

AND

('Autopsy'/exp OR 'pathology'/exp OR pathology:lnk OR Autopsy:ti OR Autopsies:ti OR Pathology:ti)

AND

('epidemiology'/exp OR epidemiology:lnk OR Epidemiology:ti OR Prevalence:ti OR Patterns:ti OR Cases:ti)

Web of Science Search
OPTC OR (Thyroid AND (cancer OR cancers OR carcinoma OR carcinomas OR Adenocarcinoma OR Adenocarcinomas neoplasm OR neoplasms OR nodule OR nodules OR tumor OR tumour OR Tumors OR Tumours OR cyst OR cysts))

AND

Autopsy OR Autopsies OR Postmortem OR Post-mortem OR (Post AND mortem)

AND

Prevalence OR Prevalences OR Epidemiology OR Epidemiological OR Frequency

© 2021 LeClair et al. *JAMA Intern Med.*

AND

Incidental OR Unsuspected OR Discovery OR Discoveries OR Findings OR Finding OR Occult OR Hidden

Web of Science Title Search (for Finding Articles Before 1975)
((Thyroid AND (cancer OR cancers OR carcinoma OR carcinomas OR Tumor OR Tumors OR Tumor OR Tumours OR Neoplasm OR Neoplasms)))

AND

(Autopsy OR Autopsies OR Pathology)

AND

(Epidemiology OR Prevalence OR Patterns OR Cases)

© 2021 LeClair et al. *JAMA Intern Med.*

eFigure 1. Preferred Reporting Items for Systematic Reviews and Meta-Analyses (PRISMA) Flow Diagram of Study Selection for Quantitative Synthesis

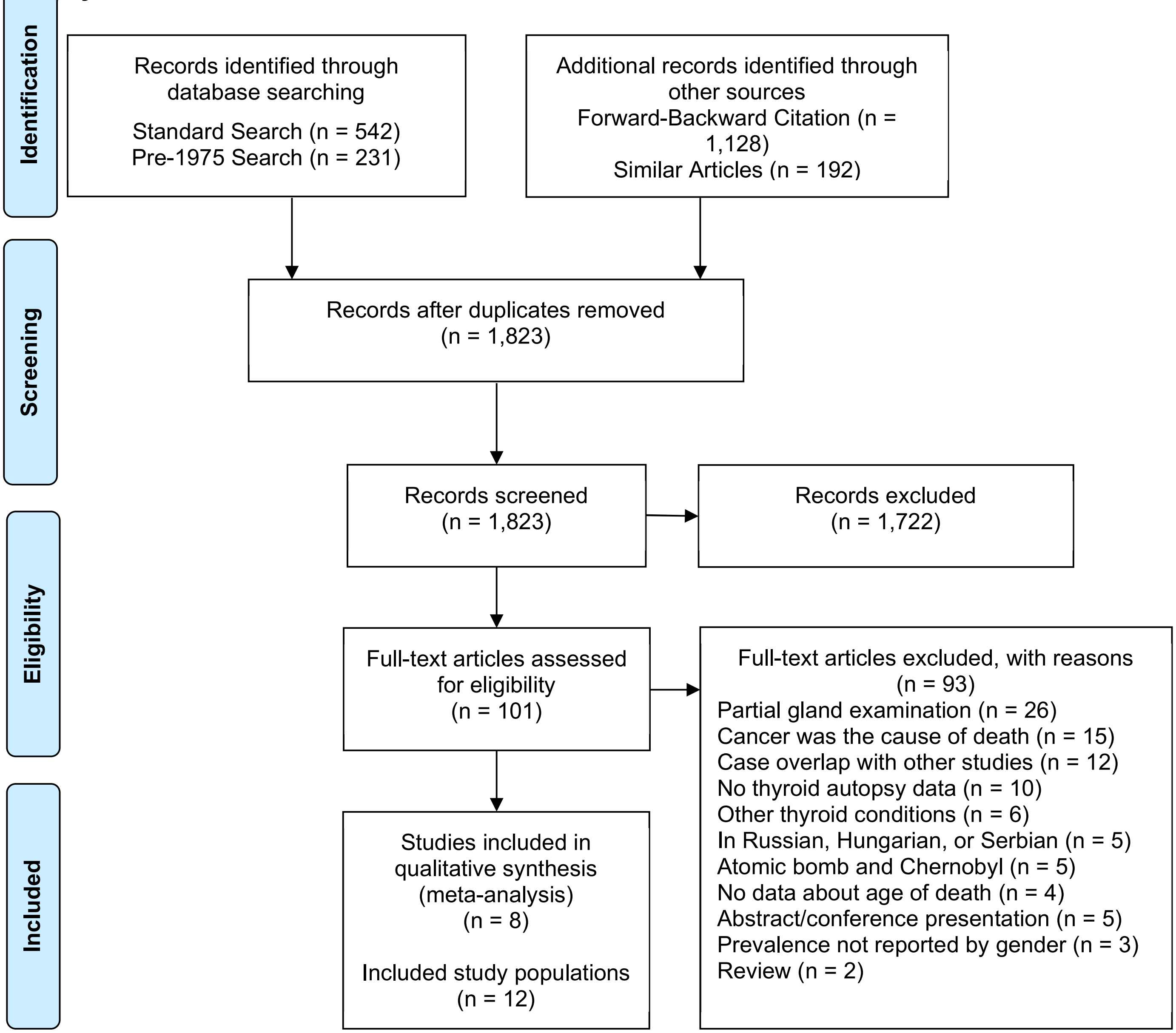

© 2021 LeClair et al. *JAMA Intern Med.*

eTable 2a. Incidence per 100,000 people for thyroid cancer during 1975 – 2017, SEER 9 Data.						
	**All thyroid cancer**		**Papillary: <2cm and localized**		**Papillary: all other sizes and stages**	
**year**	**women**	**men**	**women**	**men**	**women**	**men**
1975	6.45	3.12				
1976	6.56	2.93				
1977	7.32	3.5				
1978	6.95	3.14				
1979	6.17	2.68				
1980	6.15	2.39				
1981	6.24	2.52				
1982	6.15	3				
1983	6.51	2.8	1.79	0.57	2.03	0.9
1984	6.94	2.65	1.79	0.36	2.37	0.92
1985	7.09	3.1	1.9	0.59	2.22	1.09
1986	7.49	3.07	1.81	0.55	2.79	1.13
1987	7.12	2.81	1.94	0.57	2.5	0.93
1988	6.88	2.94	1.89	0.43	2.41	1.05
1989	7.66	2.99	2.29	0.56	2.76	1.09
1990	7.96	2.93	2.57	0.56	2.9	1.05
1991	7.69	3.19	2.45	0.6	2.82	1.21
1992	8.12	3.52	2.75	0.64	2.68	1.36
1993	7.64	3.59	2.44	0.63	2.78	1.2
1994	8.76	3.39	2.99	0.66	3.04	1.26
1995	9.02	3.37	3.14	0.67	3.25	1.27
1996	9.52	3.49	3.21	0.81	3.53	1.19
1997	9.81	3.66	3.49	0.81	3.61	1.35
1998	10.12	3.72	3.86	0.82	3.73	1.51
1999	10.67	3.87	4.24	0.82	3.62	1.69
2000	11.11	4.05	4.15	1	4.13	1.71
2001	12.19	4.34	5.01	1.13	4.5	1.75
2002	13.43	4.95	5.75	1.45	5.01	2.11
2003	14.5	4.72	6.17	1.44	5.4	1.88
2004	14.94	5.24	6.48	1.65	5.75	2.19
2005	15.98	5.81	7.4	1.82	5.82	2.37
2006	16.65	5.86	7.91	1.63	6.01	2.63
2007	18.54	6.12	9.18	1.87	6.59	2.75
2008	19.73	6.57	9.22	2.13	7.47	2.91
2009	21.59	7.09	10.45	2.34	8.16	3.39
2010	21.07	6.66	9.84	2.2	8.66	3.05
2011	21.96	7.41	10.45	2.58	8.6	3.54
2012	21.81	8.05	10.36	2.69	8.94	3.98
2013	22.4	7.78	10.78	2.57	9.18	3.9

© 2021 LeClair et al. *JAMA Intern Med.*

eTable 2a continued. Incidence per 100,000 people for thyroid cancer during 1975 – 2017, SEER 9 Data.						
	All thyroid cancer		Papillary: <2cm and localized		Papillary: all other sizes and stages	
year	women	men	women	men	women	men
2014	22.39	7.72	10.5	2.27	9.36	3.96
2015	22.3	7.73	10.12	2.19	9.64	4.14
2016	21.4	7.59	10.02	2.31	8.94	3.96
2017	19.72	7.55	9.35	2.22	8.1	4.02

© 2021 LeClair et al. *JAMA Intern Med.*

eTable 2b. Mortality per 100,000 people for thyroid cancer during 1975 – 2017. National Center for Vital Statistics data (1975 – 2017, all thyroid cancer) and SEER Incidence Based Mortality data (1992-2017, papillary thyroid cancer).

	all thyroid cancer		papillary thyroid cancer	
**year**	**women**	**men**	**women**	**men**
1975	0.65	0.39		
1976	0.61	0.49		
1977	0.63	0.46		
1978	0.61	0.44		
1979	0.58	0.43		
1980	0.53	0.38		
1981	0.55	0.42		
1982	0.53	0.37		
1983	0.5	0.34		
1984	0.52	0.38		
1985	0.48	0.39		
1986	0.49	0.39		
1987	0.48	0.4		
1988	0.45	0.41		
1989	0.44	0.41		
1990	0.48	0.37		
1991	0.46	0.38		
1992	0.5	0.38	0.18	0.09
1993	0.5	0.39	0.16	0.15
1994	0.44	0.38	0.18	0.19
1995	0.44	0.41	0.14	0.18
1996	0.47	0.42	0.2	0.14
1997	0.46	0.45	0.11	0.15
1998	0.47	0.38	0.19	0.13
1999	0.45	0.44	0.19	0.18
2000	0.48	0.47	0.21	0.25
2001	0.48	0.47	0.19	0.21
2002	0.48	0.45	0.2	0.22
2003	0.46	0.43	0.18	0.15
2004	0.47	0.47	0.14	0.22
2005	0.49	0.48	0.29	0.15
2006	0.51	0.46	0.19	0.22
2007	0.48	0.51	0.18	0.28
2008	0.52	0.5	0.24	0.18
2009	0.52	0.51	0.19	0.22
2010	0.51	0.5	0.22	0.15
2011	0.49	0.52	0.21	0.2
2012	0.46	0.51	0.23	0.26

© 2021 LeClair et al. *JAMA Intern Med.*

eTable 2b continued. Mortality per 100,000 people for thyroid cancer during 1975 – 2017. National Center for Vital Statistics data (1975 – 2017, all thyroid cancer) and SEER Incidence Based Mortality data (1992-2017, papillary thyroid cancer).				
	**all thyroid cancer**		**papillary thyroid cancer**	
**year**	**women**	**men**	**women**	**men**
**2013**	0.5	0.53	0.22	0.3
**2014**	0.5	0.49	0.19	0.27
**2015**	0.48	0.54	0.25	0.27
**2016**	0.52	0.55	0.27	0.25
**2017**	0.47	0.52	0.26	0.31

© 2021 LeClair et al. *JAMA Intern Med.*

eTable 3. Quality assessment of the included studies in the meta-analysis

Author, year	External validity				Internal validity				
	The autopsy service received subjects that were a close representation of the national population	The autopsy service did not deliberately restrict study subjects in any way (e.g., age, gender, etc.) except for previous history of thyroid disease	Some form of random selection was used or a census (e.g., consecutive subjects) - select the subjects	Non-availability of data was <20% among the selected subjects	Data collected directly from the histopathology (not autopsy notes)	An acceptable case definition was used for DTC (must have stated criteria)	The cancer detection method was reliable and valid (i.e. whole gland with fine slices examined)	Same mode of thyroid examination for all subjects in the study	Numerator and denominator match the reported results
Fukunaga and Yatani 1975	N	N	N	N	Y	Y	Y	Y	Y
Harach et al. 1985	N	Y	Y	Y	Y	Y	Y	Y	Y
Komorowski and Hanson 1988	N	N	N	Y	Y	Y	Y	Y	Y
Martinez-Tello et al. 1993	N	Y	N	Y	Y	Y	Y/N	N	Y
Neuhold et al. 2001	N	Y	Y	Y	Y	Y	Y	Y	Y
Ottino et al. 1989	N	Y	Y	Y	Y	Y	Y	Y	Y
Seta and Takahashi 1976	N	Y	Y	N	Y	N	Y	Y	Y
Thorvaldsson et al. 1992	Y	N	Y	Y	Y	Y	Y	Y	Y

© 2021 LeClair et al. *JAMA Intern Med.*

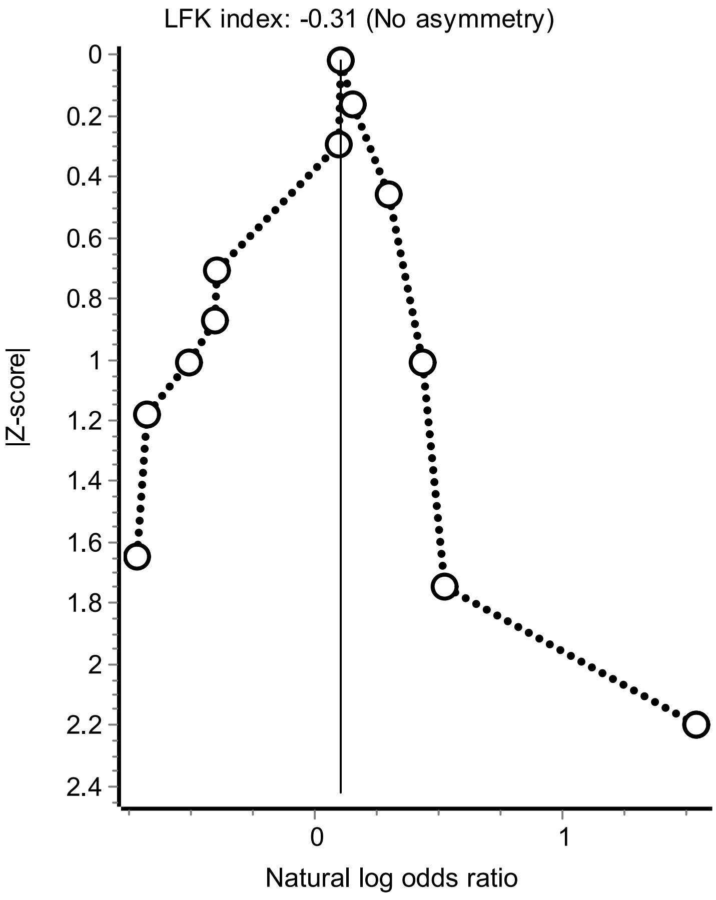

eFigure 2: Doi plot and LFK index for assessment of publication bias.

© 2021 LeClair et al. *JAMA Intern Med.*
